# Dissection of iron signaling and iron accumulation by overexpression of subgroup Ib bHLH039 protein

**DOI:** 10.1038/s41598-017-11171-7

**Published:** 2017-09-07

**Authors:** Maria Augusta Naranjo-Arcos, Felix Maurer, Johannes Meiser, Stephanie Pateyron, Claudia Fink-Straube, Petra Bauer

**Affiliations:** 1 Institute of Botany, Heinrich-Heine University, D-40225 Düsseldorf, Germany; 2 Department of Biosciences-Plant Biology, Saarland University, D-66123 Saarbrücken, Germany; 3 Transcriptomic Platform, Institute of Plant Sciences - Paris-Saclay, Plateau du Moulon, 91190 Gif-sur-Yvette, France; 4 Leibniz Institute for New Materials gGmbH, Campus D2.2, 66123 Saarbrücken, Germany; 5 Cluster of Excellence on Plant Sciences (CEPLAS), Heinrich-Heine University, D-40225 Düsseldorf, Germany

## Abstract

Iron is an essential growth determinant for plants, and plants acquire this micronutrient in amounts they need in their environment. Plants can increase iron uptake in response to a regulatory transcription factor cascade. *Arabidopsis thaliana* serves as model plant to identify and characterize iron regulation genes. Here, we show that overexpression of subgroup Ib bHLH transcription factor bHLH039 (39Ox) caused constitutive iron acquisition responses, which resulted in enhanced iron contents in leaves and seeds. Transcriptome analysis demonstrated that 39Ox plants displayed simultaneously gene expression patterns characteristic of iron deficiency and iron stress signaling. Thereby, we could dissect iron deficiency response regulation. The transcription factor FIT, which is required to regulate iron uptake, was essential for the 39Ox phenotype. We provide evidence that subgroup Ib transcription factors are involved in *FIT* transcriptional regulation. Our findings pose interesting questions to the feedback control of iron homeostasis.

## Introduction

Adequate plant nutrition with iron is critical for plant development and harvest. The presence of this metal cofactor is essential for multiple biochemical electron transfer reactions in all cells. At the same time, this metal may cause deleterious effects and oxidative stress due to radical formation through the Fenton reaction. Since the bioavailability of iron varies with soil types and is mostly rather low, plants are challenged to adapt and develop an efficient iron acquisition system. Two different strategies are distinguished for iron acquisition in plants. These strategies allow plants to mobilize and take up the amounts of iron they need also under conditions of low iron supply^1^. Strategy I, found in plants except in grasses, is based on the uptake of ferrous iron upon soil acidification and iron reduction, aided by the exudation of small organic compounds like phenolics, riboflavin or acids^2^. Strategy II, on the other side, present in grasses, relies on the uptake of ferric iron chelated by mugineic acid-based phytosiderophores^3^. Both strategies are regulated at the transcriptional level by networks of iron-regulated transcription factors^2, 3^. Attempts are underway to characterize the major players of iron regulation by using model plant systems. The knowledge about iron uptake regulation will enable us to design novel strategies for targeted breeding of iron-efficient crops.

Three types or subfamilies of bHLH transcription factors control iron uptake in dicotyledonous plants. The bHLH proteins FER and FER-LIKE IRON DEFICIENCY-INDUCED TRANSCRIPTION FACTOR (FIT) are encoded by single genes and are essential for iron acquisition in tomato and Arabidopsis^4–7^. While FER/FIT are a specific Strategy I component, the other bHLH subgroups are present in Strategy I and II plants. The bHLH subgroup Ib comprises bHLH038, bHLH039, bHLH100 and bHLH101 in Arabidopsis and the rice homolog IRO2^8–11^. The bHLH subgroup IVc is represented by POPEYE (PYE), bHLH104, IAA-LEUCINE RESISTANT3 (ILR3/bHLH105) and bHLH034 in Arabidopsis and the rice homolog IRO3^12, 13^.

FIT acts in the root and up-regulates the genes encoding IRON-REGULATED TRANSPORTER1 (IRT1) and FERRIC REDUCTASE OXIDASE2 (FRO2), two major players for ferrous iron uptake in the Strategy I^4, 5, 7, 14, 15^. Coumarins aid to mobilize iron in the soil and their production and export are also under control of FIT as well as some other root cell metal homeostasis functions^16–19^. *FIT* is induced by iron deficiency. The *FIT* gene is regulated at transcriptional level, which involves FIT itself 11. *FIT* overexpression in plants is not sufficient to promote iron uptake^5, 20^. FIT requires protein activation upon iron deficiency. At least in part this is mediated by FIT protein-protein interaction and stability control, that serve to fine-tune iron acquisition in response to hormone signals and abiotic stress factors^21, 22^. FIT was found in an active state, when *FIT* was overexpressed together with any one of the four *BHLH* subgroup Ib genes^23, 24^.

The bHLH subgroups Ib and IVc are part of a regulatory cascade leading to the onset of iron deficiency responses. *BHLH* genes from the subgroup Ib and *PYE* from the subgroup IVc are co-expressed in Arabidopsis across different plant growth conditions. These *BHLH* genes are up-regulated by iron deficiency along with genes encoding regulators, transporters and enzymes with iron homeostasis functions in shoots and roots, for example, BRUTUS (BTS), FERRIC REDUCTASE OXIDASE3 (FRO3), NATURAL RESISTANCE-ASSOCIATED MACROPHAGE PROTEIN4 (NRAMP4), OLIGOPEPTIDE TRANSPORTER3 (OPT3) and NICOTIANAMINE SYNTHASE4 (NAS4) 16. Subgroup IVc proteins can interact with each other and with the E3 ligase BRUTUS (BTS)^12, 25–27^. BTS is discussed to be an iron/metal sensor since its stability is affected by the binding of iron/metal cofactors^26, 28^. bHLH104, ILR3 or bHLH034 can bind directly to promoters of *BHLH* subgroup Ib genes and activate them^25, 27^. Loss of function of bHLH IVc factors comes with an increased sensitivity to alkalinity-mediated or general iron deficiency, while overexpression causes iron uptake^25, 27^. *BHLH101* overexpression was able to partially rescue the iron deficiency phenotype of *bhlh34 bhlh104* mutants by promoting *IRT1*, *FRO2* and *FIT* expression^25^. Thus, these findings suggest that bHLH IVc factors act upstream of bHLH Ib and FIT transcription factors. Single knockout mutants of the four subgroup Ib *BHLH* genes did not have any impact on plant growth 11. Double and triple mutants showed different degrees of leaf chlorosis phenotypes upon iron deficiency. On the other hand, no phenotypic differences were apparent under sufficient iron supply^8, 23, 29^. Generally stronger leaf chlorosis and growth phenotypes were found in those double and triple mutants, in which a *bhlh039* knock-out was present^23^. Transcriptome analysis has shown that a subset of FIT target genes was not up-regulated in response to iron deficiency in *bhlh039 bhlh100 bhlh101*, but among them were not *FRO2* and *IRT1* 8. These results support that bHLH038, bHLH039, bHLH100 and bHLH101 are not fully functionally redundant.

From these observations the idea can be put forward that FIT is not only activated at protein level upon iron deficiency, but that *FIT* gene expression is induced downstream of the iron regulatory bHLH transcription factor cascade. Here, we present evidence for this idea by studying iron accumulation caused by bHLH039 overexpression. FIT was found essential for this phenotype, and transcriptomic data analysis gave hints on the hierarchy of regulatory responses upstream and downstream of this subgroup Ib bHLH protein.

## Results

### Physiological phenotypes of bHLH39 overexpression (39Ox) plants in response to iron

Previous reports indicated that subgroup Ib bHLH proteins are active in plant and yeast cells when overexpressed together with FIT^23, 24^. However, we show here that overexpression of the subgroup Ib protein bHLH039 in Arabidopsis even without overexpression of FIT can lead to significant differences in iron (Fe) acquisition responses. We have generated stable transgenic Arabidopsis lines expressing *BHLH039* behind the double cauliflower mosaic virus 35 S promoter and tagged with a sequence encoding the hemagglutinine (HA) antigen for protein immunodetection. Root growth was diminished upon sufficient iron supply (+Fe) in four independent lines, when compared to the wild type (Suppl. Figure 1A). In the wild type, *BHLH039* was induced upon iron deficiency in both roots and shoots compared to the Fe sufficiency situation (Suppl. Figure 1B,C), as expected^11^. In the four transgenic lines, *BHLH039* was expressed at higher level than in the respective wild type samples, showing that the transgene caused an over-expression of *BHLH039* in leaves and in roots upon + and −Fe (Suppl. Figure 1B,C). Line JM78-5 with high *BHLH039* expression was selected for detailed physiological and molecular analysis. This line was designated as 39Ox. In this line, HA-bHLH039 protein was present at +Fe and −Fe in roots and leaves of two week-old plants (Suppl. Figure 1D). Hence, we can deduce that bHLH39 protein was both present and functional at + and −Fe.

**Figure 1:**
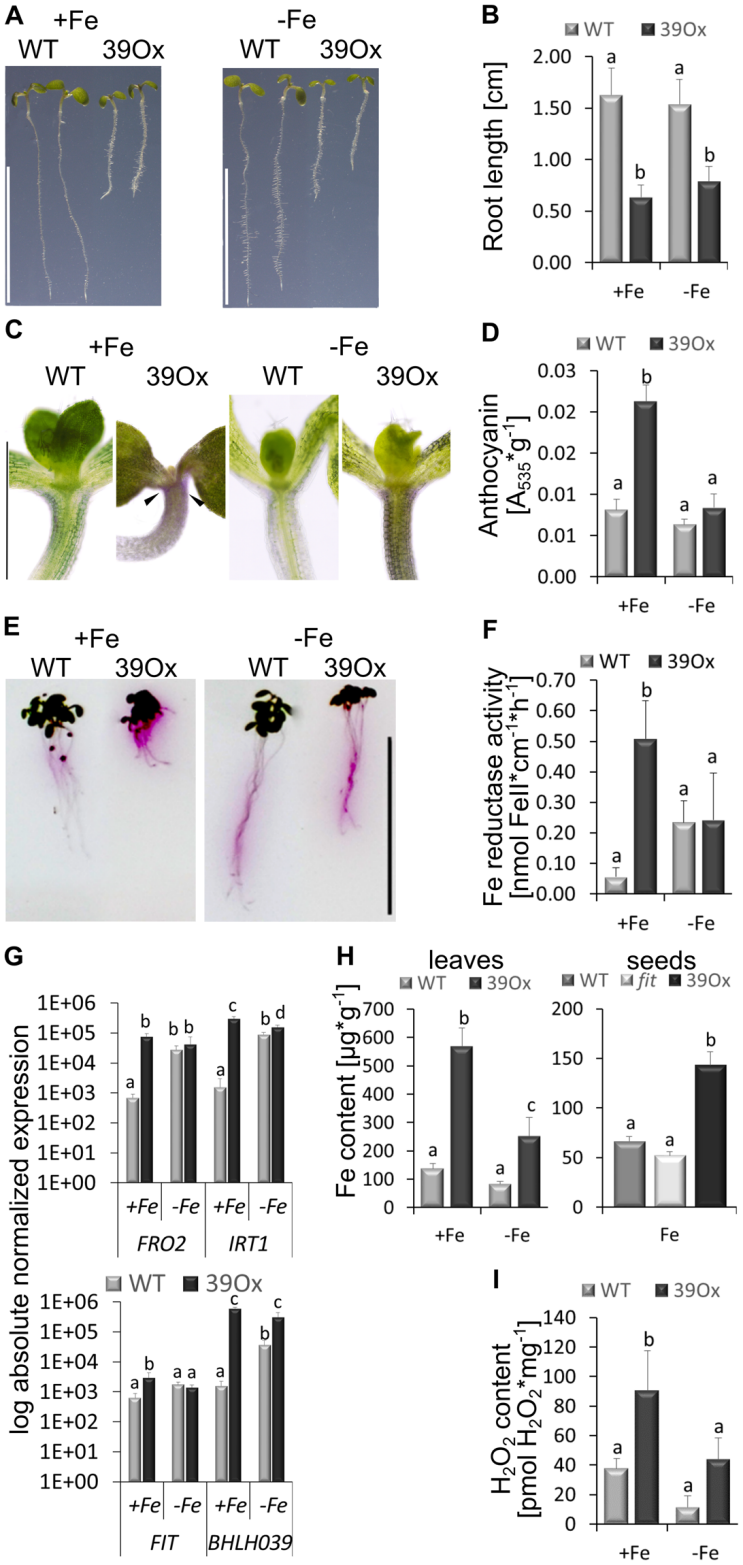
Iron response phenotypes of 39Ox plants. 39Ox and wild type (WT) seedling plants were grown under Fe sufficiency (+Fe) or deficiency (−Fe) in the six-day (**A**–**F,I**) or two-week agar plate assay (**H** left side). (**A**) Whole seedling growth (bar 1 cm); (**B**) Root lengths (n = 20); (**C**) Anthocyanin pigmentation; in the hypocotyl indicated by arrowheads (bar 1 mm); (**D**) Anthocyanin contents of seedlings (n = 3); (**E**) Visualization of root iron reductase activity (bar 1 cm); (**F**) Root Fe reductase activity (n = 4); (**G**) Seedling gene expression of iron deficiency genes *FRO2, IRT1, FIT* and *BHLH039* (n = 3); (**H**) Iron contents of leaves (left) and seeds (right) (n = 3); (**I**) Hydrogen peroxide contents of seedlings (n = 3). Error bars represent standard deviations. Different letters indicate significant differences between samples (p < 0.05).

At first, we investigated whether the short root growth phenotype caused by bHLH039 overexpression was related to iron supply. 39Ox plants were grown in a six day and in a two week growth system along with wild type plants as the control, exposed to + and −Fe and examined for iron response phenotypes. As described above, six day-old 39Ox seedlings exhibited shorter roots than the wild type upon + and −Fe (Fig. 1A,B). 39Ox seedlings had dark cotyledons and red hypocotyls when exposed to +Fe, indicating stress symptoms and anthocyanin production (Fig. 1C). Red pigmentation was not found in in wild type (Fig. 1C). Anthocyanin measurements confirmed that more anthocyanins were present at + than at −Fe in 39Ox and more in 39Ox than in the wild type at +Fe (Fig. 1D).

At −Fe, Arabidopsis plants typically display an enhanced iron reductase activity in the root to mobilize external iron. Root Fe reductase activity was increased in +Fe-grown 39Ox plants compared to the wild type, as shown by qualitative and quantitative measurements (Fig. 1E,F). The two lines had similar levels of Fe reductase activity upon −Fe (Fig. 1E,F). Fe reductase activity was higher at + than at −Fe in the 39Ox plants, perhaps caused by the lack of a negative Fe regulation. Due to the high standard deviations, the differences between + and −Fe in the wild type were not found to be significant. When we analyzed the six-day seedlings at the molecular level, we found that the Fe deficiency marker genes *FRO2* and *IRT1* were expressed at higher level in 39Ox than in wild type at +Fe, conform with the iron reductase activity data (Fig. 1G). On the other hand, at −Fe, *FRO2* and *IRT1* were rather similarly expressed in 39Ox and wild type. In the wild type, *FRO2* and *IRT1* expression were induced at - versus +Fe, which was not the case in 39Ox (Fig. 1G). *FIT* was also up-regulated in 39Ox compared to wild type at +Fe (Fig. 1G). *BHLH039* expression was found about 100 times increased at +Fe and about tenfold at −Fe in 39Ox compared to the wild type, demonstrating again the overexpression effect (Fig. 1G). Interestingly, *FIT*, *FRO2* and *IRT1* were hardly expressed in 39Ox leaves in either Fe supply condition. On the other hand, *BHLH039*, was induced in wild type leaves at −Fe versus +Fe and was overexpressed in 39Ox leaves (Suppl. Figure 2A,B). *FIT*, *FRO2* and *IRT1* were highly up-regulated at +Fe in 39Ox roots, where *BHLH039* was expressed ten to 100fold higher than in the wild type (Suppl. Figure 2C,D). *BHLH038* and *BHLH100* were not expressed in the 39Ox situation neither at + nor −Fe in leaves and roots (Suppl. Figure 2B,D). Lack of *BHLH038* and *BHLH100* expression could indicate a reduced sensing of Fe deficiency in 39Ox compared to the wild type. On the other hand, *BHLH101* showed a distinct expression compared to *BHLH038* and *BHLH100*. *BHLH101* was expressed in 39Ox similarly as in the wild type at+Fe, but the expression was reduced at −Fe (Suppl. Figure 2B,D).

Because of the generally increased iron mobilization phenotypes, we suspected that 39Ox plants might have accumulated iron. Iron measurements in leaves and seeds of 39Ox plants grown under Fe sufficiency showed a two- and fourfold increased Fe content, respectively, compared to the wild type (Fig. 1H). It was then interesting to test whether 39Ox seedlings might have experienced oxidative stress caused by the elevated Fe contents. Hydrogen peroxide measurements showed that indeed, H_2_O_2_ levels were higher in 39Ox exposed to +Fe compared to all other samples (Fig. 1I).

Taken together, overexpression of bHLH039 protein resulted in high Fe acquisition and high root to shoot and seed translocation of iron in seedlings. Due to this iron accumulation, 39Ox plants developed iron toxicity-based oxidative stress symptoms.

### Comparative transcriptome profiling of 39Ox plants

As transcription factor, bHLH039 acts in a regulatory iron sensing and iron regulation cascade. We conducted a transcriptomic microarray analysis to investigate the molecular phenotypes of 39Ox in more detail and to determine how 39Ox plants responded internally to the increased bHLH039 and Fe levels. We compared gene expression between wild type and 39Ox six day-old seedlings exposed to + and −Fe. We chose the six-day growth system because we had previously collected transcriptome data sets for this condition using wild type, *fit* mutant and a triple *bhlh* knockout mutant^8, 18^. The transcriptomic study was based on the CATMA two-color microarray and included three biological replicates of the samples. Similar CATMA microarray data were recently collected with *fit* mutant and wild type^18^. Genes with at least 1.5fold statistically significant differential regulation were retained for further analysis (Suppl. Table 1).

We found that 3745 genes were differentially regulated at +Fe comparing 39Ox with wild type, comprising 1786 down-regulated and 1959 up-regulated genes. *BHLH039*, *IRT1*, *FRO2* and *FIT* were up-regulated, which confirmed the overexpression effect and the results described in the previous paragraph (Suppl. Table 1A). 1348 genes were differentially expressed between 39Ox and wild type at −Fe, comprising 813 up-regulated and 535 down-regulated genes (Suppl. Table 1B). Several Fe-regulated genes were chosen and successfully used to validate the microarray data by quantitative RT-qPCR (Suppl. Figure 3).

**Figure 2:**
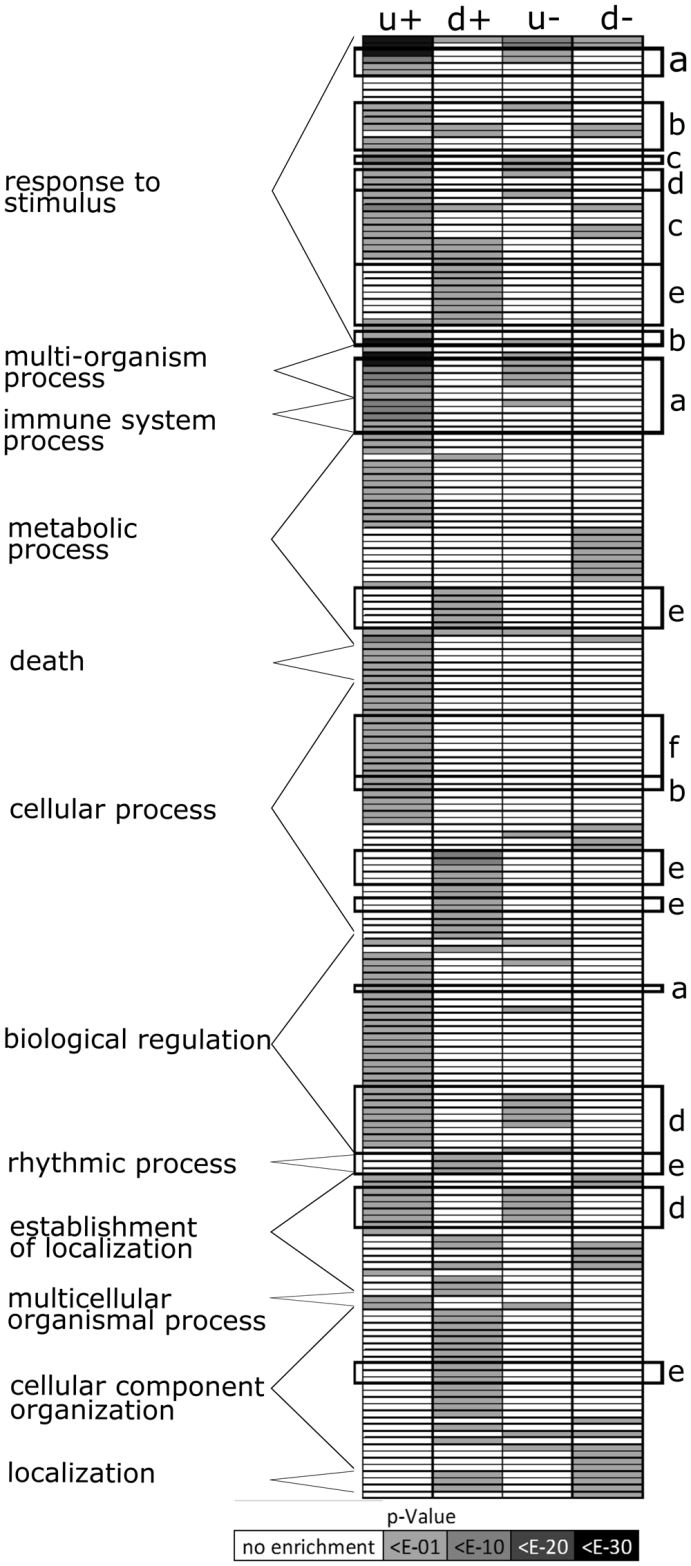
Overview of the GO term enrichment analysis of 39Ox versus wild type plant transcriptome data. The heatmap was generated based on the p-values of the up (u)- and down (d)-regulation of the GO categories in four datasets, representing pair-wise comparisons of 39Ox versus wild type under Fe sufficiency (+) and Fe deficiency (−). GO categories were enriched in at least one of the four datasets. On the left, division of GO terms according to GO level 1 descriptions. Boxes and letters on the right, accurate depiction of selected biological functions, namely a, pathogen and defense b, hormone c, abiotic and oxidative stress d, metal e, rhythm, light and photosynthesis f, organic acids. Detailed results can be found in Suppl. Table 2.

To determine which physiological and cellular pathways were affected in 39Ox based on the transcriptome profiles, we conducted a gene ontology (GO) term enrichment analysis using all genes differentially regulated in 39Ox at +Fe as well as −Fe (Fig. 2, Suppl. Table 2). Genes associated with GO categories related to stress, defense and hormone responses, regulation and metal homeostasis, secondary and cell wall metabolism and hormone biosynthesis were predominantly up-regulated in 39Ox versus wild type at + and −Fe (Fig. 2, Suppl. Table 2A,C). GO categories indicative of stress, light and hormone responses, photosynthesis, rhythm and chlorophyll biosynthesis and lipid biology were predominantly down-regulated in 39Ox versus wild type at +Fe, and to a lesser extent at −Fe (Fig. 2, Suppl. Table 2B,D). At −Fe, we also observed a down-regulation of cell wall biosynthesis and glucosinolate metabolism in 39Ox compared to the wild type (Suppl. Table 2D). The overall transcriptome data therefore indicated that 39Ox suffered from stress symptoms related to metal homeostasis.

**Figure 3:**
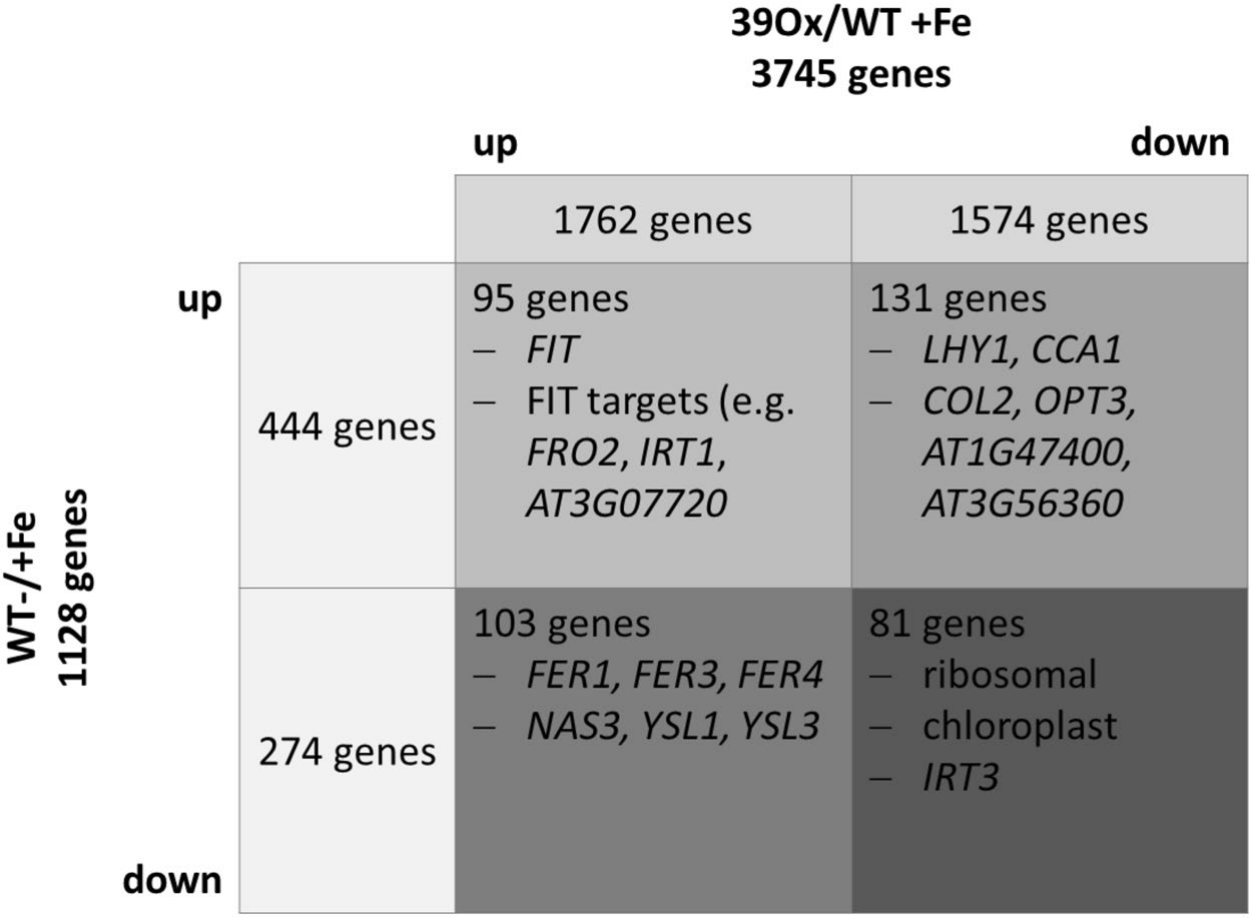
Overview of the comparison of 39Ox and Fe-regulated genes. A subset of 410 genes is contained in the intersection of 39Ox (3745 genes, this work) and Fe-regulated genes (1128 genes, 18). According to up- or down-regulation patterns, these 410 genes can be further subdivided into four groups. Represented are the numbers of regulated genes found in each group and examples of genes identified in the groups. Detailed results can be found in Suppl. Table 3.

We suspected that +Fe-grown 39Ox plants should display gene expression patterns reflecting increased iron mobilization and Fe accumulation. To check this, we compared the list of 3745 differentially expressed genes between 39Ox versus wild type at +Fe with that of 1128 Fe-regulated genes^18^ (Suppl. Table 3). We could identify an intersection of 410 regulated genes (Fig. 3, Suppl. Table 3A). 184 out of the 410 genes were down-regulated in the wild type in response to −Fe, a condition in which *BHLH039* was highly expressed in the wild type. Among them, 81 genes were also down-regulated in 39Ox, suggestive of a repression of these genes in the presence of bHLH039 protein. These 81 genes included ribosomal genes, chloroplast function-related genes and *IRT3* (Fig. 3, Suppl. Table 3A,B). However, 103 of the 184 genes were down-regulated at - versus +Fe in the wild type (down-regulated in the presence of bHLH039 at −Fe) and up-regulated in 39Ox versus wild type at +Fe (up-regulated in the presence of bHLH039 at +Fe). These 103 genes could therefore not have been regulated by bHLH039 itself but by the level of Fe. These 103 genes comprised the three ferritin genes *FER1*, *FER3* and *FER4*, typical high iron and oxidative stress marker genes^30^, and *NAS3*, *YSL1* and *YSL3*, involved in long-distance phloem-based iron translocation^31, 32^ (Fig. 3, Suppl. Table 3A). Among the 103 genes regulated in this group, a large coexpression cluster was found, related to the GO categories stress and oxidation-reduction regulation (Suppl. Table 3B). 226 out of the 410 genes were up-regulated in the wild type upon −Fe versus +Fe, when *BHLH039* was highly expressed. Among them, 95 genes were also up-regulated in the +Fe 39Ox situation, reflecting indeed an expression pattern conferred by the presence of bHLH039. These genes included −Fe-responsive genes *FIT* and FIT targets, e.g. *FRO2*, *IRT1*, *AHA7*, *MTPA2*, *At3g07720*, *MYB10*, *At3g12900*, *F6′H1*, *PDR9*, *COPT2*, *CYP82C3*, as well as Fe homeostasis genes *NAS1*, *IREG2* and *IRT2* 18 (Fig. 3, Suppl. Table 3A). 131 among the 226 −Fe-up-regulated genes were down-regulated in 39Ox, indicative of a response to Fe but not to bHLH039. These genes, being also part of several co-expression clusters, comprised several photo-oxidative stress and circadian rhythm regulators like *LHY1*, *CCA1*, *COL2*, *ELIP*. Very interestingly this list of genes also contained *NAS4* and *OPT3*, two components for phloem-based iron transport and transmission of a long-distance iron signal^32, 33^, as well as *At1g47400* and *At3g56360*, that are part of the Fe deficiency-regulated co-expression cluster of *BHLH039* 16 (Fig. 3, Suppl. Table 3A,B). Taken together, nearly one half of the iron-responsive genes differentially expressed in 39Ox versus wild type at +Fe were iron deficiency response markers, whereas more than a half of these iron-responsive genes were suggestive of a response to high internal Fe levels. Hence, 39Ox plants displayed at the same time characteristic symptoms of iron deficiency and symptoms of iron sufficiency and even iron stress. In particular, the FIT target coexpression cluster was generally induced in 39Ox at +Fe, indicative of the Fe deficiency response. On the other side, the iron homeostasis co-expression cluster containing *BHLH* Ib and IVc genes, *BTS*, *NAS4* and *OPT3*, was either not regulated or down-regulated, which reflected a high iron response of 39Ox. These patterns of 39Ox-mediated iron-related transcriptome changes were confirmed when the expression patterns of 162 robust Fe-regulated genes were studied in 39Ox (Suppl. Table3C,D).

Next, we used the transcriptome data for confirmation of downstream target genes that depend for their expression on bHLH039. Previously, 134 genes were identified, that are differentially expressed upon −Fe in six day-old seedlings of the triple knockout mutant line *3xbhlh* ( = *bhlh039 bhlh100 bhlh101*) versus wild type 8. This list of 134 genes (denoted groups I and III in ref. 8) contained 52 genes, that we found to be differentially regulated in 39Ox versus wild type. 25 out of the 52 genes were up-regulated in the *3xbhlh* and down-regulated in 39Ox versus wild type. They were mostly related to chloroplast, rhythm and photo-oxidative stress reponses in leaves (Suppl. Table 3E,F). 18 out of the 52 genes were down-regulated in *3xbhlh* and up-regulated in 39Ox. These genes comprised a large coexpression cluster and four FIT and bHLH039 potential downstream target genes, namely *At3g07720*, *At3g12900*, *CYP82C4* and *COBL6* (Suppl. Table 3E,F). Therefore, the 39Ox transcriptome data indicated again that these latter four genes might be targets regulated by bHLH039.

Finally, we investigated the regulation of^34^ FIT-dependent and root-expressed genes that we had previously described^18, 33^ of them showed differential regulation in 39Ox versus wild type (Suppl. Table 3G). 29 genes were up-regulated in the presence of FIT and in 39Ox and included the FIT-dependent iron uptake genes *IRT1* and *FRO2*. Two genes down-regulated by FIT were also found to be down-regulated in 39OX, namely *ZIP2* and *SCPL31*. Hence, the regulation of FIT downstream genes in 39Ox plants likely depended on the presence of FIT. Since FIT is an essential protein for iron uptake, the 39Ox effect is well explained by the activation of the FIT response pathway through bHLH039.

### Requirement of FIT for 39Ox phenotype

If *FIT* is indeed an important target activated downstream of bHLH039, we predicted that FIT should be required for regulation of the FIT targets and the 39Ox phenotype at +Fe.

**Figure 4:**
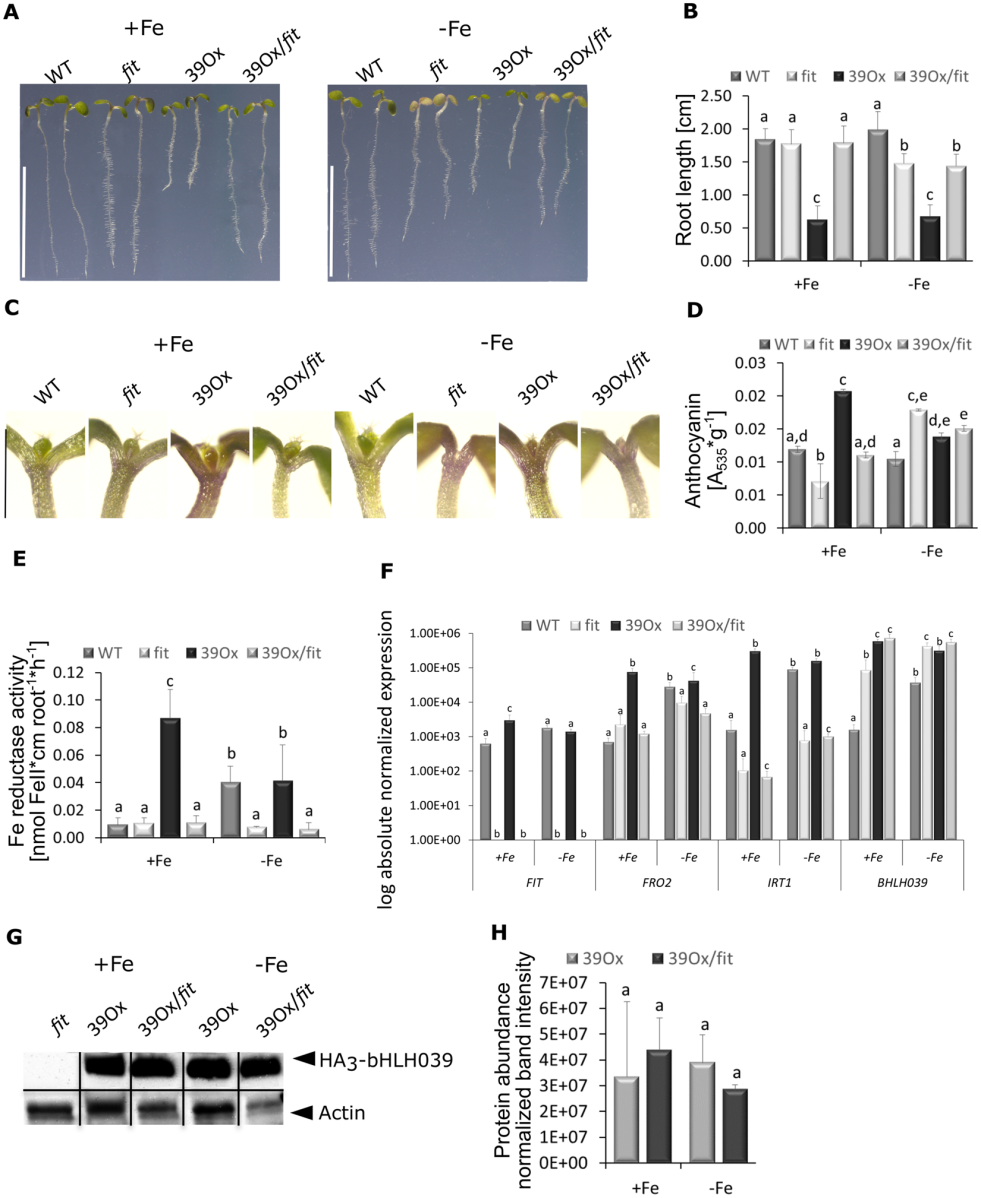
Analysis of the 39Ox effect in the *fit* mutant background. Wild type (WT), *fit* mutant (*fit*), 39Ox and 39Ox with *fit* mutant background (39Ox/*fit*) seedling plants were grown under Fe sufficiency (+Fe) or deficiency (−Fe) in the six-day agar plate assay. (**A**) Root growth phenotype (bar 1 cm); (**B**) Root lengths (n = 20); (**C**) Magnification of the shoots from (**A**) (bar 1 mm); (**D**) Anthocyanin contents of seedlings (n = 3); (**E**) Root iron reductase activity (n = 4); (**F**) Gene expression of *FIT*, *FRO2*, *IRT1* and *BHLH039* (n = 3); (**G**) Immunoblots with anti-HA antibodies and actin as a loading control. Arrowheads indicate the positions of the bands corresponding to HA_3_-bHLH039 (36 kD) and actin (42 kD). *fit* seedlings grown at +Fe were used as a negative HA-protein detection control. (**H**) Protein abundance based on immunoblot signals of HA_3_-bHLH039 normalized to actin (n = 3). Error bars represent standard deviations. Different letters indicate significant differences between samples (p < 0.05).

To test this, we investigated the 39Ox phenotype in the background of *fit* loss of function mutant plants. Such *fit* mutant plants do not mobilise sufficient iron and suffer from severe leaf chlorosis 5. We found that in contrast to 39Ox, 39Ox/*fit* plants showed similar morphological and physiological phenotypes as *fit* mutants. 39Ox/*fit* plants had leaf chlorosis and did not display the short root phenotype of 39Ox (Fig. 4A,B). Instead, 39Ox/*fit* roots grew similarly as *fit* roots. They were as long as wild type roots at +Fe, and reduced in length at −Fe (Fig. 4B). 39Ox/*fit* plants did also not have the strong anthocyanin pigmentation of 39Ox (Fig. 4C,D). However, plants with *fit* background showed more anthocyanin pigmentation than the wild type at −Fe, probably due to the iron deficiency stress. Iron reductase activity was low in 39Ox/*fit* at + and −Fe like in *fit* plants (Fig. 4C). Furthermore, gene expression analysis confirmed that *BHLH039* was overexpressed. *FRO2* and *IRT1* gene expression were low and not highly induced by −Fe nor by +Fe in 39Ox/*fit*, as it was observed in *fit* mutants (Fig. 4D). Gene expression levels of FIT-dependent genes *AT3g07720*, *At3g12900*, *MTPA2* and *CYP82C* were also comparably low in 39Ox/*fit* and *fit* mutant (Suppl. Figure 4). HA-tagged bHLH039 protein was equally expressed in 39Ox plants with wild type as well as *fit* mutant background (Fig. 4E,F). These results indicate that a functional FIT was required for the action of bHLH039 in 39Ox. FIT did not affect the protein abundance of bHLH039.

**Figure 5:**
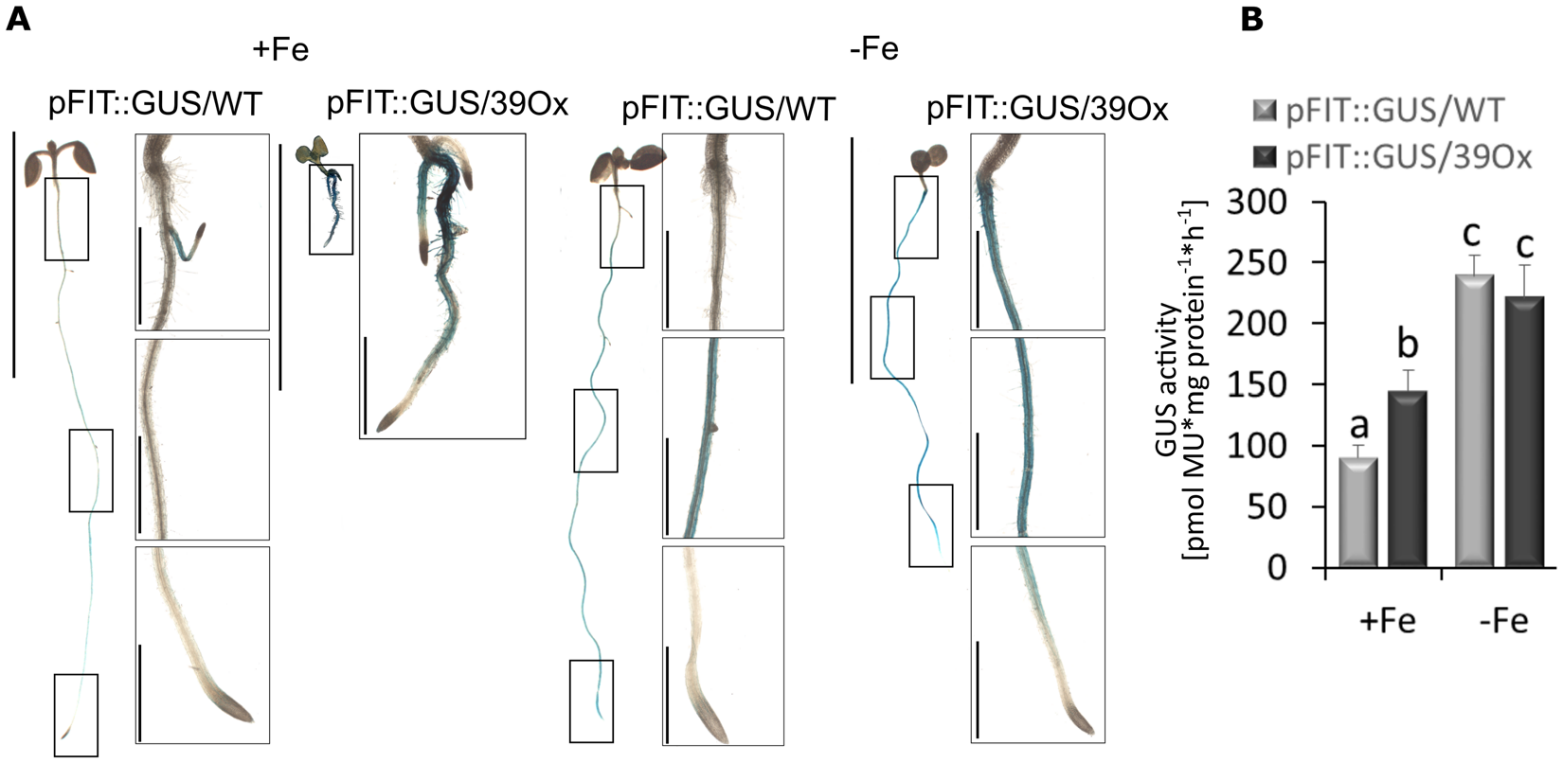
Analysis of *FIT* promoter-driven b-glucuronidase (GUS) activity. pFIT::GUS seedlings with wild type (pFIT::GUS/WT) and 39Ox mutant background (pFIT::GUS/39Ox) were grown under Fe sufficiency (+Fe) or deficiency (−Fe) in the six-day agar plate assay. (**A**) Qualitative GUS activity assay, on the left, whole plant (bar 1 cm) with squares, representing close-ups on the right (bar 1 mm). The three squares represent the transition from hypocotyl to upper root zone (top), middle zone of the root (middle), and root tips (bottom). (**B**) Quantitative GUS activity assay (n = 3). Error bars represent standard deviations. Different letters indicate significant differences between samples (p < 0.05).

Above, it was shown that Fe reductase activity was found along the roots of 39Ox seedlings. Thus, we suspected that *FIT* expression might also have been activated all along the root in response to 39Ox. To investigate in which region of the root the *FIT* gene was activated, we localized *FIT* promoter activity based on *FIT* promoter-driven beta-glucuronidase reporter activity in transgenic pFIT::GUS plants with 39Ox background. In wild type background, *FIT* promoter activity was detected mainly in the epidermis cells of the elongation root zone in Fe-deficient conditions, as expected 5 (Fig. 5). GUS activity was stronger in plants with 39Ox than wild-type background at +Fe (Fig. 5B). GUS activity was detected along the entire 39Ox roots, but not in hypocotyls and cotyledons (Fig. 5A). Thus, overexpression of bHLH039 activated the *FIT* promoter in an ectopic manner in roots.

## Discussion

Here we showed, by using overexpression plants and combinations of transgenic and mutant plants, that bHLH039 induces directly or indirectly *FIT* in the Fe deficiency regulatory cascade and that bHLH039 is active even in the presence of sufficient iron.

A remarkable conclusion from this study is that bHLH039 protein was not only abundant in 39Ox plants but also functionally active regardless of Fe supply. On the contrary, FIT and HA-FIT were present at + and −Fe in plant cells in an overexpression situation, but only active at −and not +Fe^5, 20^. It was known that *BHLH039* is manifold induced at −Fe, while at +Fe hardly any transcripts are produced. In contrast, the *FIT* gene is expressed at +Fe and induced about threefold at −Fe 5. Hence, the action of bHLH039 is regulated in the plant mainly via gene expression regulation, while the action of FIT is regulated at transcriptional and protein level. The transcriptional activation of *BHLH039* makes sense in view of the current models about the iron deficiency regulatory cascade. According to these models gene expression of *BHLH039* and of other *BHLH* subgroup Ib genes is induced by subgroup IVc bHLH factors in response to −Fe^25, 27^.

The most striking 39Ox phenotypes were apparent at +Fe but not at −Fe, showing that the utilization of Fe was affected in the 39Ox plants. First, the 39Ox plants displayed high expression of iron acquisition genes, high Fe reductase activity, high Fe contents in leaves and seeds and high ferritin gene expression, in contrast to the wild type. These phenotypes clearly show that 39Ox plants took up more Fe from the growth medium than the wild type. Second, 39Ox plants displayed oxidative stress symptoms at +Fe, like up-regulation of oxidative stress gene expression, anthocyanin pigmentation, short root phenotypes and H_2_O_2_ production. Both Fe accumulation and oxidative stress phenotypes of 39Ox were Fe-dependent. We interpret the observed 39Ox phenotypes as follows (depicted in a model in Suppl. Figure 5): The transcription factor bHLH039 up-regulated together with low levels of FIT protein the *FIT* gene. FIT and bHLH039 then induced iron acquisition genes like *FRO2* and *IRT1*. The resulting FRO2 activity and IRT1 stimulated iron uptake into the roots. Iron was translocated to shoots and high amounts of Fe accumulated in leaves and seeds. The high Fe levels in roots and shoots then caused toxicity symptoms such as oxidative stress, short root growth and anthocyanin pigmentation. At least half of the Fe-regulated differentially expressed genes in 39Ox versus wild type were indicative of a high iron response that normally serves to alleviate iron stress. For example, ferritin *FER* gene expression was increased, together with *NAS3*, *YSL1* and *YSL3*. These genes were up-regulated in response to the high Fe status in the shoot, indicating that iron was bound and/or circulated in the plant. Ferritin was presumably used to suppress oxidative stress and to store Fe in plastids^30–32^. Even though these protective responses were enhanced, we need to assume that they were not sufficiently effective to alleviate the iron stress in 39Ox. Surprisingly, repressive iron signals, that normally would be expected to delimit excessive iron uptake, were switched off or ineffective in +Fe-grown 39Ox plants. It has been hypothesized that BTS could be a sensor of Fe and negative regulator in the transcription factor cascade^12, 26, 28^. BTS protein function and regulation in plant cells in response to cellular Fe awaits further experimentation 26. *BTS* was not up-regulated in 39Ox and therefore BTS could not likely have repressed the cascade. Perhaps low BRUTUS (BTS) E3 ligase protein levels or levels of related proteins were active in 39Ox and effectively suppressed the Fe deficiency cascade at the level of subgroup IVc bHLH proteins. *BHLH* IVc genes are not Fe-regulated, with the exception of *PYE* that we did not detect in our list of 39Ox up-regulated genes either. Moreover, very interestingly, *BHLH038*, *BHLH100* and *BHLH101* were down-regulated in 39Ox at + and −Fe. Since subgroup IVc bHLH proteins induce the promoters of *BHLH* Ib genes, we suspect that the Fe deficiency cascade upstream of *BHLH* subgroup Ib genes was switched off^25, 27^. Furthermore, four genes linked with that cascade and normally induced by −Fe and co-expressed with *BHLH039*, namely *OPT3*, *NAS4*, *At1g47400* and *At3g56360*, were even down-regulated in 39Ox. Overexpression of bHLH039 must have counteracted any suppressor effects by BTS or related proteins. Our results are therefore in agreement with previous published data, such as overexpression of a bHLH subgroup IVc protein leading to iron over-accumulation phenotypes similar as seen in 39Ox or *bts* loss of function plants^25, 27^. A long distance iron suppressor signal transported via the phloem from shoot to root has been hypothesized to communicate the shoot iron status to the root^11, 32, 34, 35^. A long-distance iron signal was suspected to be loaded into the phloem of leaf cells via OPT3^33, 34^. Such a long-distance signal was either inactive in 39Ox or in case it acted at the level of the transcription factor cascade upstream of bHLH039 not effective in 39Ox. The down-regulation of *OPT3* in 39Ox could reflect a potential mechanism for the inhibition of transmission of a long distance iron suppressor signal from leaf to root. However, it could also be that OPT3 may not be involved in the transmission of a signal. Finally, local suppressor mechanisms for iron uptake are described that suppress the activity of individual proteins mediating iron uptake in the root epidermis cell. For example, IRT1 localization is altered in the presence of metals^36^. Under sufficient Fe, IRT1 is more prone to degradation^37^. FRO2 is described to be inactive upon sufficient Fe despite of the presence of this protein in a *FRO2* overexpression situation^38^. It is surprising that these negative regulation mechanisms acting inside root epidermal cells were also not in place in the 39Ox background. This suggests that protein regulation factors for IRT1 and FRO2 might be related to the action of FIT.

bHLH039 action required functional FIT. 39Ox/*fit* plants lost the Fe-dependent 39Ox phenotypes. Therefore, FIT must have been functionally active in 39Ox plants, and again, potential inhibitory components previously reported to negatively control FIT protein activity^20^ were not present or in place in 39Ox. This gives support to the idea that the main activator of FIT protein in 39Ox was bHLH039. The same occurs in wild type plants exposed to −Fe (see model in Suppl. Figure 5). Once subgroup Ib bHLH factors are produced in root cells in response to the regulatory Fe deficiency cascade, FIT becomes activated at protein level through direct protein interactions, as shown^23, 24^. FIT was found to be involved in its own feed-forward gene expression regulation^11^. In 39Ox plants, the *FIT* gene promoter was activated in an ectopic manner in roots but not in leaves. Hence through the interaction with bHLH039 in 39Ox plants, FIT might have further stimulated its own transcription all along the root. This scenario can also explain why molecular Fe acquisition responses like *IRT1* and *FRO2* induction were only switched on in roots but not in leaves of 39Ox. FIT is a root-specific transcription factor 5 and hence a basal level of FIT was required at +Fe for bHLH039 action in 39Ox. Different scenarios of direct or indirect positive bHLH039-mediated *FIT* promoter induction are conceivable. One possibility is that bHLH039-FIT heterodimers bind to the *FIT* promoter (direct activation of *FIT* promoter by FIT-bHLH039 heterodimers, evidence for FIT heterodimerization in^24^). Alternatively, FIT may bind to its promoter as a homodimer being activated by binding in addition to bHLH039 (direct activation of *FIT* promoter by multiprotein complexes consisting of FIT homodimers and bHLH039, evidence for FIT homodimerization in^24^). Finally, an additional yet unknown transcription factor could be induced by bHLH039 and FIT and then serve to activate the *FIT* promoter and FIT target genes (indirect regulation of *FIT* promoter by FIT and bHLH039). High Fe stress-related anthocyanin production was not found in 39Ox leaves with *fit* mutant background. Hence, such shoot responses could not have been under direct or indirect control of bHLH039. Therefore, anthocyanin production was more likely the result of secondary effects related to iron accumulation in the shoot rather than the action of bHLH039 in the cells.

In conclusion, bHLH039 is a direct or indirect activator of *FIT* transcription in the root. Any suppressive Fe signals were inactive in 39Ox and did not inhibit Fe uptake. By manipulation of bHLH039 expression, it was thus possible to enhance iron levels in shoots and seeds. Our findings raise interesting questions with regard to the coupling of iron signals with post-translational negative control mechanisms acting upon iron uptake components. An interesting question to pursue in the future is to tackle the roles of bHLH subgroup Ib factors in the shoot. Since the Fe levels of 39Ox leaves and seeds were enhanced, it is conceivable that bHLH039 was involved in regulating the delivery of Fe to sinks.

## Methods

### Plant material and growth conditions

The wild type ecotype used was Col-0. *fit-3* (termed *fit* throughout the text) and pFIT::GUS plants were previously described^5^.

bHLH039 overexpression plants were constructed as follows: A genomic DNA fragment containing exons and introns of *BHLH039* was inserted into pALLIGATOR yielding a double cauliflower mosaic virus 35S promoter 2xpCaMV::*triple hemagglutinine (HA) tag-gBHLH039* coding cassette, according to a previously described procedure^20^. Transgenic lines were generated, genotyped and multiplied until homozygous lines were obtained. Four lines were investigated and one line selected for further analysis (here designated 39Ox), as described in the text (see Suppl. Figure 1). 39Ox/*fit-3* (termed 39Ox/*fit*) and pFIT::GUS/39Ox plants were generated after crossing and selection of homozygous lines.

For experiments, seeds were surface sterilized and plants raised in a six-day agar plate assay in the presence of 50 microM Fe (+Fe) or 0 Fe (−Fe) or in a two-week agar plate assay with a three-day exposure to medium containing 50 microM Fe (+Fe) or 50 microM ferrozine and 0 Fe (−Fe) using a modified Hoagland medium, as previously described 5,22 and as indicated in the text and figure legends.

### Morphological and physiological plant analysis

Seedlings grown on agar plates were pictured with the Axio Zoom.V16 Stereo-Zoom-Microscope (Zeiss, Jena, Germany). Root lengths were measured after photography with the help of the ImageJ software tool.

Anthocyanin extracts were prepared from 15 preweighted seedlings by incubation for 5 minutes at 95° Celsius and then overnight at 25° Celsius with a propanol:HCl:H_2_O (18:1:81 v/v/v) mixture, as decribed^39^. The extract was photometrically measured at OD535. After background subtraction (OD_650_) the values were normalized to the respective sample weights.

Iron reductase activity was determined using a quantitative liquid and qualitative agar plate assay with ferrozine as indicator for Fe^2+^, as previously described^40^. Quantitative iron reductase activity was normalized to root length.

For iron content determination, leaves were harvested, dried, powdered and processed for graphite furnace atomic absorption spectroscopy, as previously described^22^. Seeds were dried, weighed and microwave-digested (Multiwave 3000, Anton Paar GmbH, Graz, Austria) with concentrated HNO_3_. Fe concentrations were determined by inductively-coupled plasma-optical emission spectrometry ICP-OES with a Czerny-Turner type monochromator (Ultima 2, Horiba Jobin-Yvon, New Jersey, USA, and Longjumeau, France).

Hydrogen peroxide contents were measured using the Amplex red hydrogen peroxide/peroxidase assay kit (Molecular Probes) as previously described^21^.

### beta-glucuronidase (GUS) assay

For localization of GUS activity, pFIT::GUS and pFIT::GUS/39Ox plants were incubated in the GUS staining buffer (50 mM sodium phosphate, 2 mM potassium ferrocyanide, 2 mM potassium ferricyanide, 0.2% Triton X-100, and 2 mM GUS substrate 5-bromo-4-chloro-3-indolyl-b-D-glucuronic acid) according to^41^. Leaf chlorophyll was removed by incubation in 70% ethanol.

Quantitative GUS activity analysis using total protein extract was conducted as previously described, following a fluorescence measurement method with MUG substrate (4-methylumbelifetyl-beta-D-glucuronide)^5^.

### Gene expression analysis by RT-qPCR

A detailed protocol of the RT-qPCR procedure is available^40, 42^. RNA from about 60 seedlings per biological replicate was extracted. cDNA was synthetized with poly dT primer from DNase-treated total RNA (300–500 ng) and then analyzed by real time qPCR using the DyNAmo ColorFlash SYBR Green qPCR Kit (Thermo Scientific, Waltham, USA) and the ICycler (Bio-Rad, Hercules, USA), as described^42^. Primers and primer sequences are listed in^8, 18^. qPCR data accuracy and reliability was checked as described^40, 42^. The initial transcript amount of two technical replicates was determined by standard curve analysis using mass standards and averaged. The absolute expression data were normalized with the constitutive expression control gene encoding elongation factor 1Bα1 (*EF1B*α*1*). The mean of three biological replicates per sample was calculated and used for statistical treatment of RT-qPCR data.

### Microarray-based transcriptome analysis

Six-day wild type and 39OX seedlings were raised on +Fe an −Fe in three biological replicates. 60 seedlings were pooled per sample and RNA extracted using the Qiagen plant RNA extraction kit (Qiagen, Hilden, Germany). A two-color CATMAv6 microarray analysis was performed including a dye-swap control, following the published procedure^43^. After the statistical analysis gene lists were retained with log_2_ normalized intensities per samples, log_2_ normalized ratios, and p-values (FDR correction). Probes with a Bonferroni p-value of 0.05 or less and a fold change of 1.5 or more were considered differentially expressed. The same RNA preparations were also used for validation by RT-qPCR of selected genes.

Transcriptome data sets were compared using Excel. Gene ontology (GO) term and coexpression analysis was conducted and visualized using VirtualPlant^44^, Revigo^45^ and STRING^46^, as indicated in the text and legends.

### Immunoblot analysis

Immunoblot analysis was conducted according to^21^. Around 40 six day-old seedlings were harvested, frozen in liquid nitrogen and grinded using the Precellys (PeqLab Biotechnologie GmbH, Erlangen, Germany). Per 1 mg plant powder 1 microl of 2x Laemmli buffer was added. After centrifugation 10 microg heat-denatured total protein was loaded on a 12% SDS-polyacrylamide gel and proteins separated by electrophoresis. Proteins were electro-transferred to a nitrocellulose membrane (GE Health Care, Amersham) with transfer buffer. HA_3_-bHLH039 protein was detected after immunoblotting with anti-HA-horseradish peroxidase (HRP)-coupled high-affinity monoclonal rat antibody (3F10 clone 1:1.000 dilution; Roche Diagnostics GmbH, Basel, Switzerland) by chemiluminescence (ECL chemiluminescence kit, GE Health Care, Little Chalfont, UK) and the FluoChem Q (Biozym, Hamburg, Germany) device. The membrane was stripped and reused for the detection of actin protein with anti-actin antibodies (rabbit anti-ACT, 1:5000 dilution; Agrisera, Vännäs, Sweden) and goat anti-rabbit-HRP-coupled secondary antibody (Pierce, 1:1000 dilution; Thermo Fisher Scientific, Carlsbad, USA).

The intensities of the bands were determined using the software AlphaView (FluorChem Q). The local backgrounds were subtracted. HA signal intensity was normalized to that for actin. Alternatively and as mentioned in the text and figure legend, normalization was conducted to major bands observed and measured after Ponceau S staining of the membrane for total protein.

### Statistical analysis

Statistical analysis was conducted using the one-way ANOVA test followed by the post-hoc Tukey test for pair-wise comparisons. p-values of 0.05 or lower indicate statistical significance between two mean sample values, represented in the figures by different letters. p-values above 0.05 indicate no statistically significant difference between the two compared sample values, indicated by the same letter above the two sample values in the figures. The diagrams represent the mean values of biological replicates and the error bars reflect standard deviations.

## Electronic supplementary material

Supplementary Figures

Suppl. Table 1

Suppl. Table 2

Suppl. Table 3
