# Supplementary material for: Dissection of iron signaling and iron accumulation by overexpression of subgroup Ib bHLH039 protein
Source: Sci Rep. 2017 Sep 7;7:10911. doi: 10.1038/s41598-017-11171-7 (PMC5589837; doi:10.1038/s41598-017-11171-7)
Supplement: Supplementary file 1 — Supplementary Figures [file 41598_2017_11171_MOESM1_ESM.pdf]

Supplemental Figures File

Dissection of iron signaling and iron accumulation by overexpression of subgroup Ib bHLH039 protein

Maria Augusta Naranjo-Arcos, Felix Maurer, Johannes Meiser, Stephanie Pateyron, Claudia Fink-Straube, and Petra Bauer

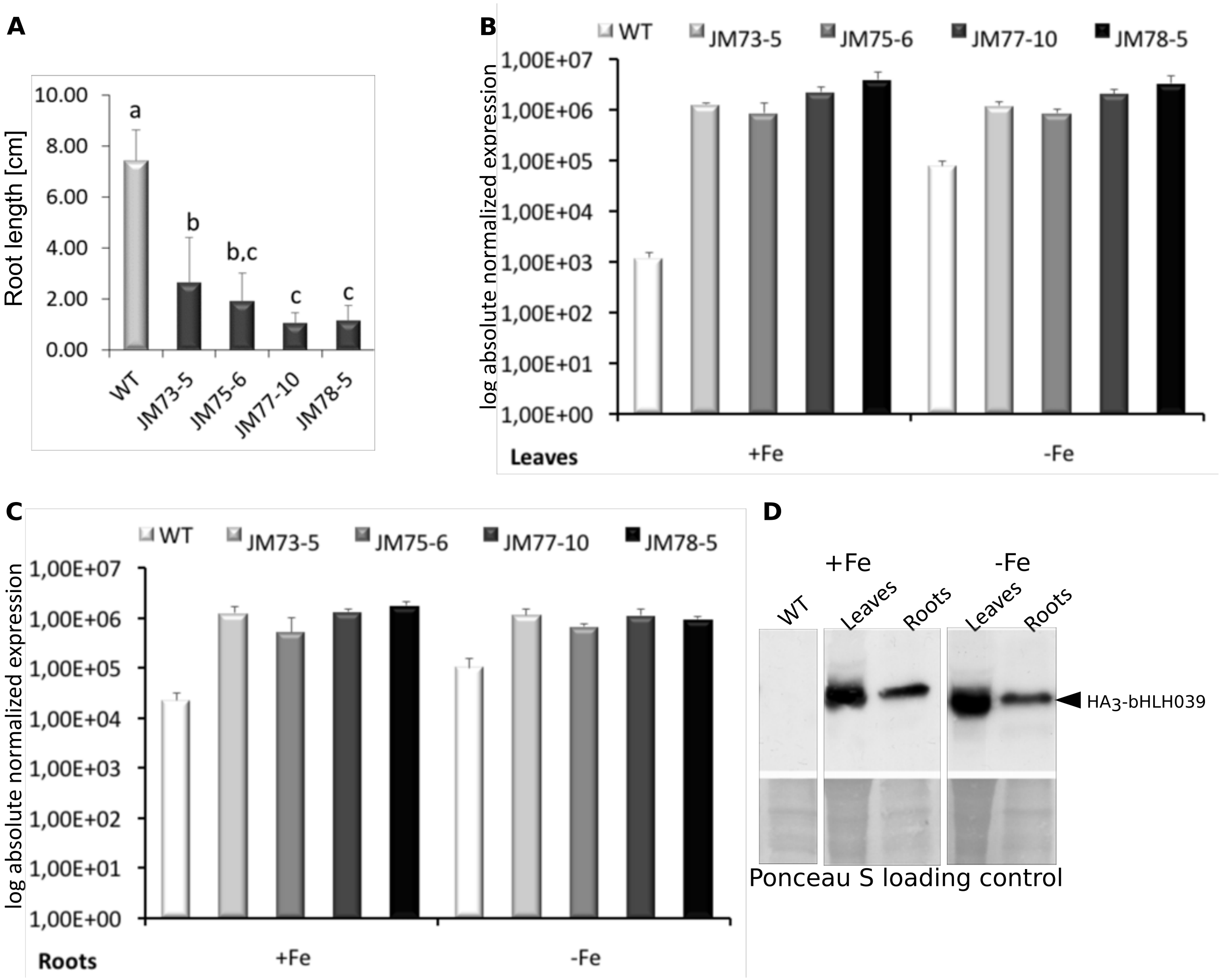

Suppl. Figure 1. Initial characterization of four bHLH039 overexpression lines. The lines were JM73-5, JM75-6, JM77-10 and JM78-5. JM78-5 was selected for further analysis and was termed throughout this work 39Ox. Plants were grown in the two-week agar plate assay. A, Root lengths (n= 15-50). Gene expression of *BHLH039* in B, leaves and C, roots (n=2). D, Immunoblot with anti-HA antibodies and Ponceau S staining as a loading control. The arrowhead indicates the positions of the bands corresponding to HA_3-bHLH039 (36 kD). WT plants grown at +Fe were used as a negative HA-protein detection control. Error bars represent standard deviations. Different letters indicate significant differences between samples ($p < 0.05$).

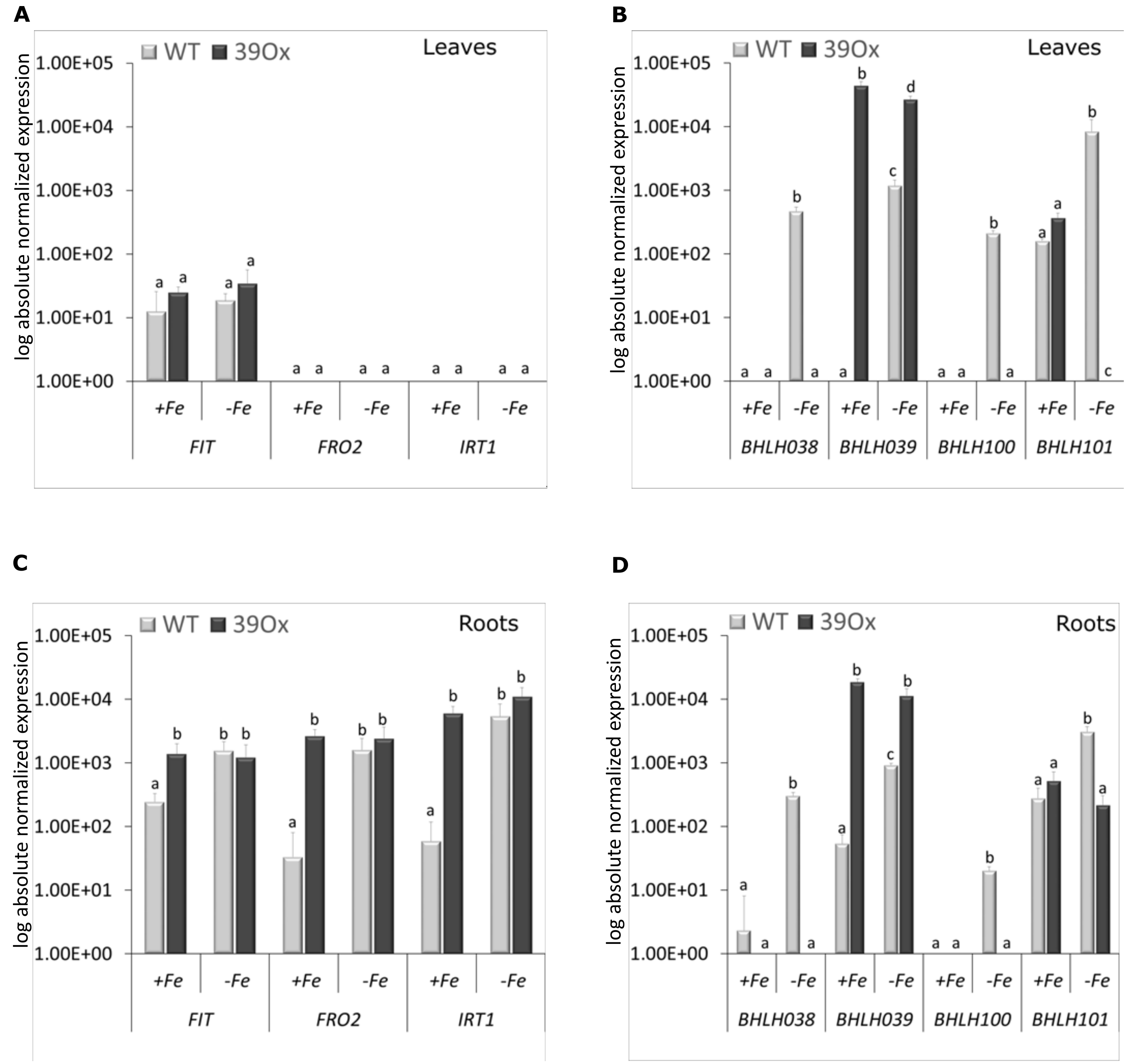

Suppl. Figure 2. Gene expression in leaves and roots of 39Ox plants. 39Ox and wild type (WT) seedling plants were grown under Fe sufficiency (+Fe) or deficiency (-Fe) in the two-week agar plate assay.
A, C, Gene expression of *FIT*, *FRO2* and *IRT1*, and B, D, of *BHLH038*, *BHLH039*, *BHLH100* and *BHLH101* in A, B, leaves and C, D, roots. Error bars represent standard deviations. Different letters indicate significant differences between samples ($p < 0.05$, $n = 3$).

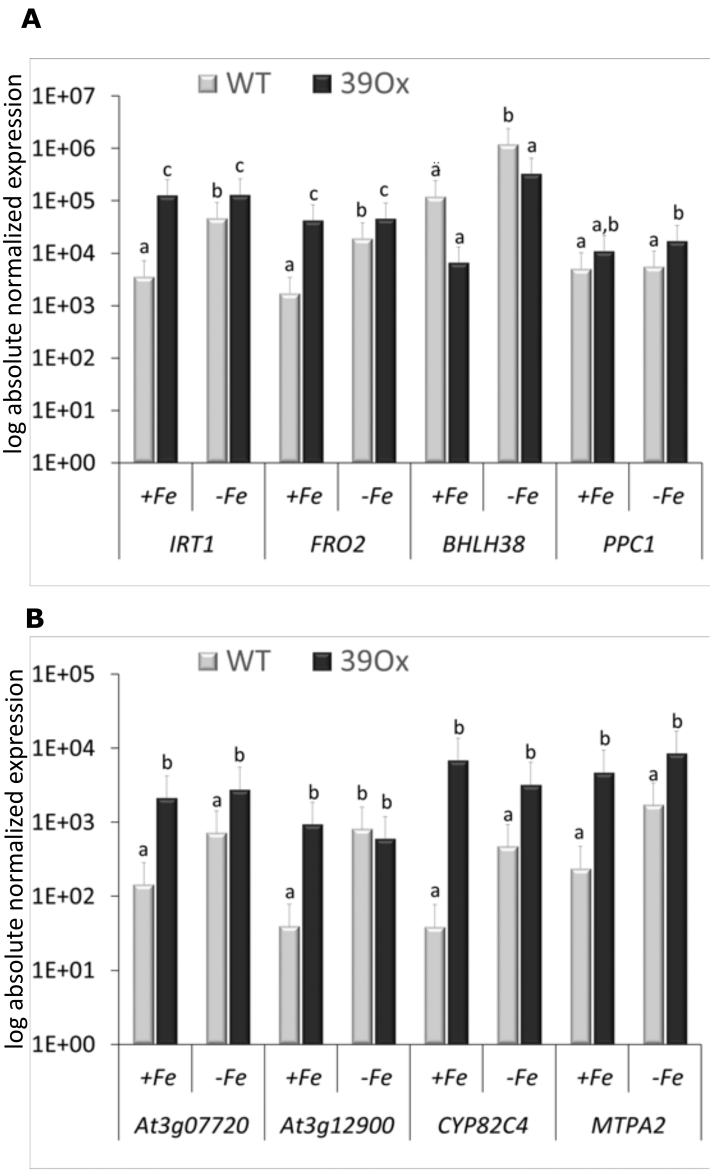

Suppl. Figure 3. Validation of microarray gene expression data by RT-qPCR. 39Ox and wild type (WT) seedling plants were grown under Fe sufficiency (+Fe) or deficiency (-Fe) in the six-day agar plate assay. A, gene expression of *IRT1*, *FRO2*, *BHLH038*, *PPC1* and B, of *AT3g07720*, *At3g12900*, *CYP82C4* and *MTPA2*. Error bars represent standard deviations. Different letters indicate significant differences between samples ($p < 0.05$, $n = 3$).

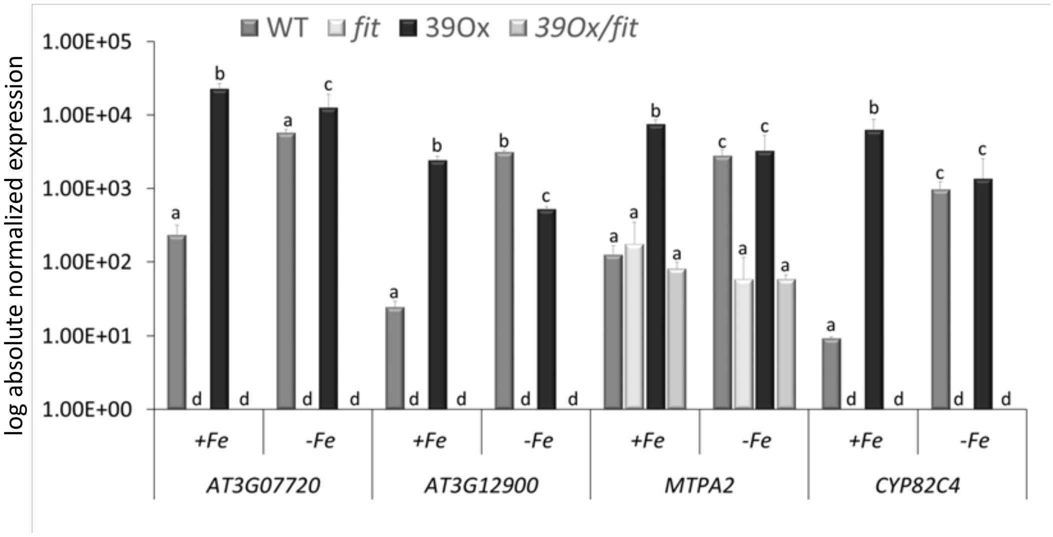

Suppl. Figure 4. Analysis of gene expression in 39Ox with *fit* mutant background. Seedling plants were grown under Fe sufficiency (+Fe) or deficiency (-Fe) in the six-day agar plate assay. Gene expression of *AT3g07720*, *At3g12900*, *MTPA2* and *CYP82C4*. Error bars represent standard deviations. Different letters indicate significant differences between samples ($p < 0.05$, $n = 3$).

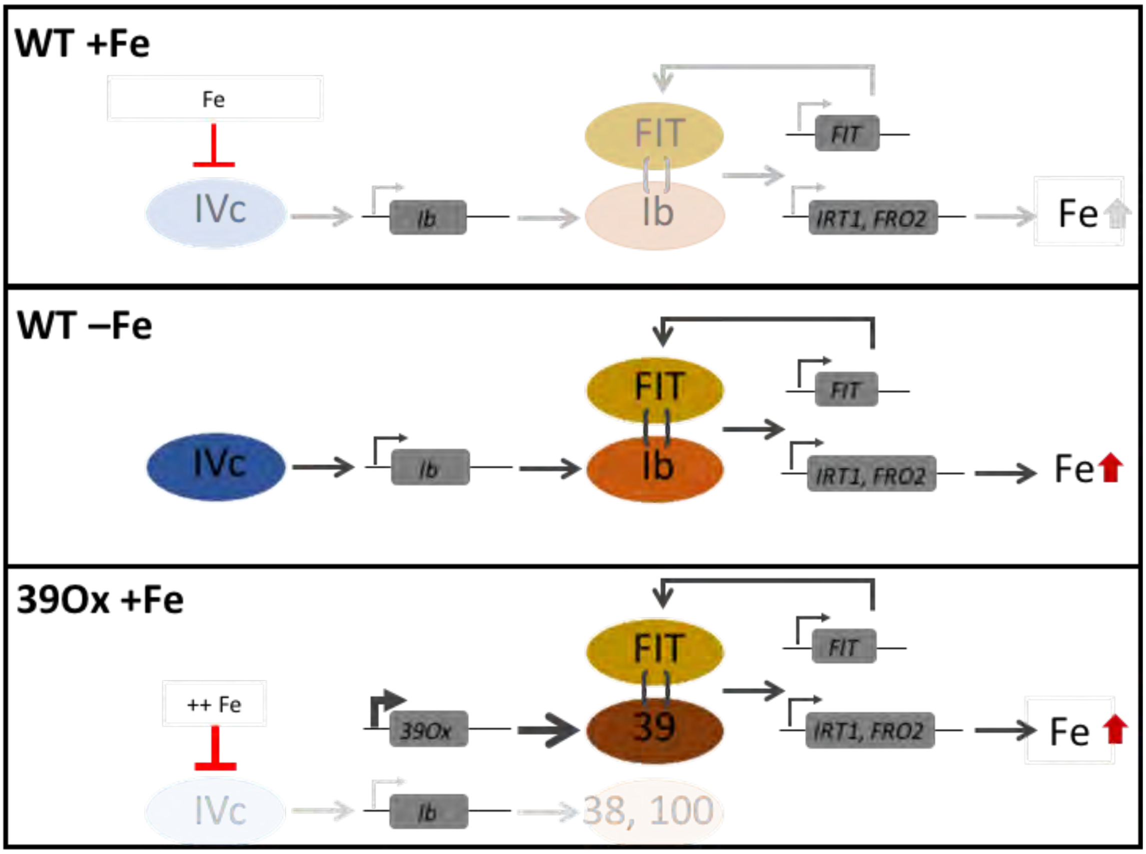

Suppl. Figure 5. Model showing the regulation of iron uptake in wild type and 39Ox plants.
WT + Fe (+ Fe-grown wild type plants), iron is available and taken up as needed with low activities of IRT1 and FRO2. The cellular Fe leads to a repression of bHLH subgroup IVc transcription factors (see discussion in the text). Consequently, *BHLH* subgroup Ib genes (comprising *BHLH038*, *BHLH039*, *BHLH100*, *BHLH101*) are expressed at low level and bHLH Ib transcription factors are present in low amounts. This results in low interaction of bHLH subroup Ib and FIT. Hence, *FIT*, *IRT1* and *FRO2* genes remain expressed at low level.
WT − Fe (− Fe-grown wild type plants), low cellular Fe levels result in an activation of bHLH IVc transcription factors, which then induce gene expression of *BHLH* subgroup Ib genes. Subgroup Ib bHLH transcription factors stimulate upon their interaction with FIT the expression of *FIT*, *IRT1* and *FRO2* genes. This results in higher levels of FIT protein, IRT1 and FRO2. Root cells thus have higher capacities for Fe uptake.
39Ox + Fe (+ Fe-grown 39Ox plants), the *BHLH039* transgene is highly expressed. The resulting bHLH039 proteins interact with FIT, thereby stimulate transcription of *FIT*, *IRT1* and *FRO2*. High levels of FIT, IRT1 and FRO2 protein in the presence of iron cause high Fe uptake levels and Fe accumulation in the plants. The high cellular Fe levels result in oxidative stress responses (see discussion in the text). High cellular Fe levels cause inhibition of bHLH IVc transcription factors and consequently strongly reduced expression of *BHLH* subgroup Ib genes *BHLH038* and *BHLH100* and respective proteins.

The symbols represent: ovals, proteins; grey squares, genes; corner arrows, respective gene expression; square brackets, protein-protein interaction; arrows, enhancement; inhibitory symbol, repression. Low levels are indicated by 80 %, very low levels by 95 % color transparency.

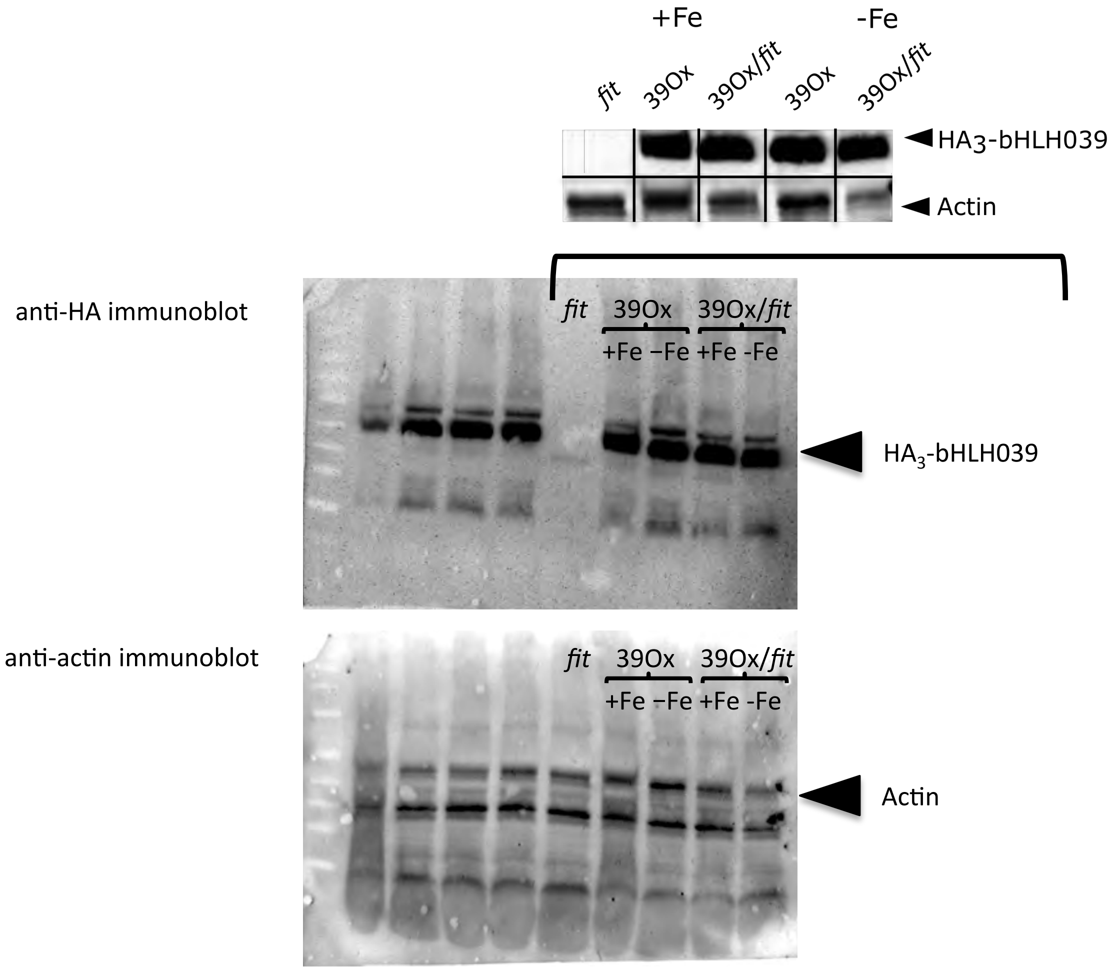

Supplementary information. Uncropped and unprocessed original scans of immunoblot data of Figure 4G, Immunoblots with anti-HA antibodies and anti-actin as a loading control (reproduced at the top). Arrowheads indicate the positions of the bands corresponding to HA_3-bHLH039 (36 kD) and actin (42 kD). The relevant lanes are labeled with sample names.
